# Early Life Experiences Moderate the Relationship Between Genetic Risk of Autism and Current and Lifetime Mental Health

**DOI:** 10.3389/fpsyt.2021.772841

**Published:** 2021-11-30

**Authors:** Su Hyun Shin, Cheryl Wright, Susan Johnston

**Affiliations:** ^1^Department of Family & Consumer Studies, University of Utah, Salt Lake City, UT, United States; ^2^Department of Special Education, University of Utah, Salt Lake City, UT, United States

**Keywords:** mother relationship, autism genetics, police encounters, psychiatric problem, early life experience

## Abstract

Although individuals with autism are at greater risk of mental health challenges than others, we know little about the relationship between the mental health of older adults (50+) and autism because they are less likely to be diagnosed. Identifying the risk and protective factors that are associated with mental health can increase educational awareness, inform clinical practice, and provide information to help diagnose and treat older adults with autism. This study used longitudinal panel data of the 2008–2016 waves of the Health and Retirement Study. It estimated individual random-effect models by interacting a genetic propensity toward autism and early life experiences to test whether the latter has a moderating effect on the relationships between genetics and the Center for Epidemiologic Studies Depression (CES-D) score, self-reported depression, and history of psychiatric problems. Results suggest that individuals with a higher genetic propensity for autism are less likely to develop psychiatric problems if they report a positive maternal relationship early in life. Further, a combined effect of police encounters early in life and genetic risk for autism is associated with higher CES-D scores, increased odds of self-reported depression, and a history of psychiatric problems. Clinical applications of these findings include the need to establish and support high-quality relationships by addressing both child and caregiver needs. Further, these findings support the need to design and implement proactive interventions to teach police and autistic individuals how to successfully navigate these encounters.

## Introduction

Autism describes a combination of challenges in social communication, repetitive behaviors, restricted interests, and sensory sensitivities starting in early childhood (1). Recent systematic reviews and meta-analyses focusing on autism provide compelling support for a link between social communication challenges and mental health issues (2–4). Specifically, those with autism may experience social rejection, loneliness (5), difficult relationships, and the absence of friendships, all of which can lead to depression, social anxiety, and other mental health challenges (6, 7). Negative interpersonal experiences such as scarce social support, few friends, and bullying are associated with an increased level of depression among children with autism (5, 6, 8). Indeed, an increased risk of mental health challenges is supported by the data, ranging from 14.4 to 37% for those with autism (2, 3). This is double or triple the rate in the general population (9).

The heritability of autism has been well-established in twin studies. A population-based twin study found that 56–95% of autism phenotypes are attributable to genetic variations (10). Recent genome-wide association studies (GWAS) identified loci correlated with neurodevelopmental conditions and social skills. These can predict autism by regressing such phenotypes on each single nucleotide polymorphism (SNP) with controls and finding all available SNPs with the largest predictive power (11). The coefficients or odds ratios retrieved from regressions estimated in the GWAS served as weights to calculate an autism polygenic score (PGS). As the weighted sum of genotypes, an autism PGS provides stronger predictive power than a single risk gene (12). An autism PGS is an appropriate and relevant proxy for autism because it enables researchers to study various mental health outcomes even in the absence of autism diagnosis particularly in older adults who are less likely to have a diagnosis (13). A recent literature review provides supporting evidence that an autism PGS predicts autism (14).

The autism PGS might better predict the phenotypes of “high-risk” populations such as those with the 22q11.2 deletion syndrome (15), rare 16p11.2 sequence variants (16), and those who are placed on the far-right side of the autism PGS distribution (17). For example, a clinical sample of subjects aged 5–22 years admitted for autism assessment had higher levels of executive dysfunction, even after controlling for age and sex if they were in a high autism PGS group (17). An autism PGS might, therefore, better explain the autism phenotypes in high-risk populations if we can identify who is at risk. For this reason, the present study seeks to identify the environmental factors that relate to an increased risk of autism and its associated mental health issues.

The interpersonal therapy (IPT) diathesis-stress model provides a theoretical framework for a mechanism through which those with autism are more likely to suffer from psychiatric disorders such as depression (18). Genetic, biological, and personality factors are components of the diatheses that directly affect psychiatric problems (12). The model proposes that the combination of vulnerabilities in diatheses and interpersonal stress caused by contextual factors can lead to psychopathologies. Based on the diathesis-stress theoretical model, the present study hypothesizes that early life experiences as well as genetic and interpersonal vulnerabilities for those with autism are the root of many mental health challenges (19).

As suggested in the diathesis-stress model, contextual or environmental factors such as early life experiences are important components in the mental health of those with autism (4). Research about early traumatic experiences has found a significant relationship with psychiatric problems, especially depression (20). Traumatic events before adulthood include episodes of violence, injury, abuse, and other types of maltreatment (21). However, despite their adverse effects, little research has examined how early stressful events, such as troubled relationships, can affect mental health in individuals with autism (20, 21). Indeed, young people with autism might be exposed to increased risks of both stressful traumatic experiences and develop subsequent psychiatric problems (21). Specifically, if they struggle to engage in socially appropriate interactions due to a lack of social skills and social awareness, this may lower the likelihood of developing quality relationships and engaging in positive interactions with those at home and in the community (21). The absence of or having few personal relationships can decrease the quality of life for those with autism (22).

Given the research supporting the increased likelihood of mental health challenges among people with autism, it is important to identify risk and/or protective factors motivated by well-established theoretical frameworks. Suffering from lifelong mental health challenges such as depression can have significant negative impacts on lifetime outcomes and quality of life reflected in employment, education, and marriage (22). This study attempts to close a gap in the literature by highlighting interplays between a genetic propensity toward autism and early life experiences, either as risk or protective factors, that relate to mental health.

Over the past decade, interest in autism life course issues (e.g., beyond childhood and adolescence) has dramatically increased (23). Because of the challenges presented by an aging population, greater awareness is needed about the convergence of mid and later life issues and autism. Even so, little is known about the mental health outcomes of adults with autism aged 50 and over (13), and it remains unclear how early life experiences affect their mental health. This study investigates the mental health challenges associated with autism risk using longitudinal data of older adults that includes genetic profiles for autism.

The methodological limitations of previous studies restrict our understanding of the relationship between autism and current/lifelong mental health. Relatively few studies have focused on adults with autism, which has resulted in a decreased ability to recognize the impact of psychiatric problems over lifespans (4, 24). Further, selection bias in prior research relying on clinical samples from mental healthcare settings excludes generalizing to the full spectrum of those with autism (1, 24). Finally, a lack of longitudinal research makes it difficult to ascertain the extent to which lifetime mental health in autistic individuals varies with age and early life experiences (4, 24). Given these limitations, longitudinal studies of randomly sampled community-dwelling adults with a genetic propensity for autism would be especially useful.

To address these limitations, the present study used a nationally representative panel sample of the Health and Retirement Study (HRS) that collects data on individuals age 50 or older. The current study aims to explore whether those with a higher genetic propensity for autism have a higher risk of mental health problems and whether early life experiences can moderate such relationships. The results of this investigation can inform educational awareness, advise clinical practice, and contribute information about risk and protective factors for mental health issues that could help with diagnosis and treatment.

## Methods

### Data and Sample

The present study used a longitudinal panel dataset of the 2008–2016 waves of the Health and Retirement Study (HRS) that biennially surveys a nationally representative sample of community-dwelling older adults aged 50 or over and their spouses. Since 2008, the HRS has used consistent measures for the three mental health outcome variables, which will be described below. The HRS dataset collects the respondent's mental health history, genetic traits, and early life experiences. It also provides information that might be used as covariates, such as gender, age, education, marital and employment status, income and wealth, health, and family structure. Servais (25) offers further information about the dataset. This study is exempted from Institutional Review Boards (IRB) review because it uses a secondary dataset of the HRS administered by the Institute for Social Research (ISR) at the University of Michigan, which did receive IRB approval.

This study's sample includes 8,464 unique individuals and 34,536 observations (wave-respondents). The sample was restricted to those of European ancestry, following accepted practice in genetics (26, 27). For a genetic propensity toward autism, the study used a polygenic score (PGS), which is a weighted sum of genotypes associated with autism. The HRS uses weights from a replicated genome-wide association study (GWAS) that primarily relied on a sample of European descent. Including people from other ancestry groups would significantly lower the predictive power of the autism PGS (26, 27). The study also restricted the sample to those who responded to a psychosocial questionnaire that included questions about early life experiences. Beginning in 2006, the HRS included such a questionnaire and required 50 percent of the core interviewees, randomly selected and rotated, to participate.

### Measures

#### Polygenic Scores (PGS) for Autism

Between 2006 and 2012, as part of an Enhanced Face-to-Face Interview (EFTF), the HRS collected saliva samples from its participants (28). Specifically, in 2006 the HRS selected a random half of its core interviewees to participate in this EFTF, and in 2008 the other half was invited to participate. For 2010 and 2012, only first-time interviewees (i.e., new cohorts of those who were born between 1948 and 1959) were randomly invited to one of the two years. The HRS used a saliva sample to calculate the PGS for autism referencing genetic markers and weights from a GWAS conducted by the Autism Spectrum Disorders Working Group (11). In phase I of the GWAS using 7,387 autism cases and 8,567 controls, it found no single nucleotide polymorphism (SNP) that met the significance criteria (i.e., *p* ≤ 5 × 10^−8^). In phase II of the GWAS, it identified 6.1 and 5 percent of the genetic markers correlated with autism in two samples from the Danish iPSYCH (7,387 autism cases and 8,567 controls) and the deCODE/SEED (7,783 autism cases and 11,358 controls) data. The identified genes were *CUEDC2, PITX3, HDAC4, MACROD2, EXOC4, ANO4, EXT1*, and *ASTN2* (11). Using the identified genetic markers and weights (i.e., effect size) identified above, the HRS provides researchers with a continuous measure of an autism PGS. In the current study, the PGS for autism is standardized to have a mean of zero and a standard deviation of one.

#### Early Life Relational Quality With Mother

In the three waves (2008–2012), the HRS assessed perceived relational quality with the mother before age 18 using three items adopted from Rossi (29): “How much time and attention did your mother give you when you needed it?” “How much effort did your mother put into watching over you and making sure you had a good upbringing?” and “How much did your mother teach you about life?” Responses ranged from one (considerably) to four (not at all). To create a composite score, this study reverse-coded and averaged the three items. The average score was 3.44 (SD = 0.70). The score was then standardized (mean = 0, SD = 1) and treated as a time-invariant factor.

#### Early Life Trauma

The HRS measured each respondent's early life traumas before age 18 using the four items derived from Krause et al. (30): whether they had to repeat a year of school; whether they were in trouble with the police; whether either of their parents drank or used drugs so often that it caused family problems; and whether they were physically abused by either parent. Responses were “yes” or “no.” Four indicators were created and coded as one if their response was “yes” and zero otherwise. The proportion of the sample with each trauma was 14, 6, 20, and 9 percent, respectively (Table 1). These indicators were treated as time-invariant factors because such events happened before the respondents were 18.

**Table 1:** Sample characteristics, 2008–2016 HRS.

**Variables**	**Mean (S.D.)**	**Variables**	**Mean (S.D.)**
*Control variables*		Number of children	2.97 (1.89)
Female	0.58 (0.49)	Number of siblings	2.35 (1.96)
Age	69.83 (10.68)	Mother's premature death	0.03 (0.19)
Education		Father's premature death	0.06 (0.23)
Less than high school	0.08 (0.28)	Number of years of mother's education	10.80 (2.99)
Some college	0.26 (0.45)	Number of years of father's education	10.45 (3.51)
Bachelor's degree	0.30 (0.45)	*Outcome variables*	
Marital status		CES-D score	1.14 (1.48)
Sep./div./wid.	0.28 (0.42)	Depression	0.13 (0.24)
Never married	0.03 (0.17)	Psychiatric problem	0.17 (0.37)
Self-reported health		*Explanatory variables*	
Poor	0.05 (0.18)	Autism PGS	0.02 (0.99)
Fair	0.15 (0.27)	Relational quality with mother	3.44 (0.70)
Very good	0.37 (0.36)	Early lifetime trauma	
Excellent	0.11 (0.24)	Remained in the same class for a year	0.14 (0.35)
Smoking status		Was in trouble with the police	0.06 (0.24)
Past smoker	0.46 (0.49)	Parental drinking/drug problems	0.20 (0.39)
Current smoker	0.10 (0.29)	Parental physical abuses	0.09 (0.28)
Number of drinks	2.82 (5.81)	Early life discrimination	
Weight status		Dismissed from a job	0.04 (0.19)
Underweight	0.01 (0.09)	Not hired for a job	0.02 (0.12)
Normal	0.30 (0.41)	Denied a promotion	0.01 (0.09)
Obese	0.31 (0.42)	Prevented from moving	0.004 (0.06)
Employment status		Denied a bank loan	0.01 (0.07)
Employed	0.26 (0.39)	Mistreated by the police	0.02 (0.12)
Not working	0.06 (0.18)		
Household income	84,960 (100,478)		
Household net worth	683,447 (1,227,414)		
Health insurance	0.97 (0.13)		

*8,464 unique individuals, 34,536 observations. Unweighted estimates*.

#### Early Life Discrimination

As measures for early life discrimination that the respondents experienced between the age of 16 and 25, six items were consistently asked across the waves following Williams et al. (31): for unfair reasons, whether they had been dismissed from a job, not hired for a job, denied promotion, prevented from moving into a neighborhood because the landlord or a realtor refused to sell or rent to them, denied a bank loan, and stopped, searched, questioned, and physically threatened or abused by the police. The responses were “yes” or “no.” As time-invariant factors, we created six indicators coded as one if their response was “yes” and zero otherwise. Approximately 4, 2, 1, 0.4, 1, and 2 percent of the sample experienced the respective event early in life (Table 1).

Retrospective responses of HRS participants to identify the three factors for assessing early life experiences were used based upon a recent study indicating that subjective measures of childhood maltreatment can predict psychopathological risk (32). Further, although retrospective subjective measures have limitations, previous studies suggest that retrospective reports of adverse events of adults in early life may suffer from substantial false negatives, but not false positives (33). This indicates that such events are underreported—and underreporting leads to underestimating the effects of early life experiences on mental health. Despite potential measurement errors, we believe that our results are valid, at least due to the low risk of a type I error that incorrectly finds statistically significant correlations.

#### Mental Health

As a measure for mental health, the study used three outcomes: (1) Center for Epidemiologic Studies Depression (CES-D) scale, (2) self-reported depression, and (3) history of doctor-diagnosed psychiatric problems. The first two measures reflect *current* mental health condition. To assess the respondent's CES-D score, the HRS asked whether respondents experienced six negative feelings (i.e., felt depressed, alone, and sad, everything was an effort, sleep was restless, and could not get going) and two positive feelings (i.e., felt happy and enjoyed life) in the prior week. After subtracting the sum of the two positive-feeling indicators from the sum of six negative-feeling indicators, the score ranged from 0 to 8. The mean score was 1.14 (SD = 1.48). The second measure was self-reported depression. Since 2008, the HRS has asked *all* respondents whether they felt sad, blue, or depressed for 2 consecutive weeks or more in the previous 12 months. This measure captures their current mental health, but for a longer time horizon compared to the CES-D score. The responses were “yes,” “no,” and “did not feel depressed because of anti-depressant medication.” The study treated the third response to be equivalent to “yes.” Approximately 13 percent of the sample felt depressed during the study period. The last mental health measure represents *lifetime* conditions. The HRS asked whether a doctor had said the respondent had an emotional, nervous, or psychiatric problem. We created an indicator for psychiatric problems as one if the response was “yes” and zero otherwise. The percentage of the sample with psychiatric problems was 17 percent. The study used the three outcome variables as time-variant factors measured concurrently.

#### Covariates

In all models, we controlled for various factors known to correlate with mental health: gender, centered age at 50, education, marital status, self-reported health, smoking status, natural logarithm of the number of drinks per week, weight status, employment, natural logarithm of household income, inverse hyperbolic sine of net worth, health insurance ownership, number of children and siblings, parental premature death, parental education, and year-fixed effects. We log-transformed variables with a skewed distribution such as income and the number of drinks per week. To transform net worth with the skewed distribution and non-positive values, we used an inverse hyperbolic sine transformation (34). We also controlled for the principal component of the genetic data provided by the HRS. The HRS randomly labeled the genetic variables to control for systematic differences in genetics across ancestry groups. To reduce potential bias caused by the genetic data, the HRS recommends controlling for these variables (35). Table 1 presents the sample's characteristics in detail.

Slightly more than half of our sample were female (58%). A majority of the sample had at least a high school diploma (92%), was married or partnered (69%), reported good or better health (80%), was overweight or obese (69%), retired or not working (74%), and had health insurance (97%). Within our sample, 3 percent had a mother who had died before the age of 50, while 6 percent had a father who had deceased before 50. The sample had on average three living children and two siblings.

### Data Analysis

For all data analyses, the study used an individual-random effect model to account for time-invariant explanatory variables of autism PGS and early life experiences with panel data. For the continuous dependent variable of the CES-D score, we used linear regression models. For the other two dichotomous dependent variables, we estimated logit models. In all specifications, we clustered the standard errors at the individual-level to account for the panel structure of the data (i.e., having more than one observation within the same individual across years). The study first estimated whether a PGS for autism predicted the three mental health outcomes with and without controlling for the covariates. The study then modeled whether early life experiences had moderating effects on the relationship between a PGS for autism and mental health by including the interaction terms between a PGS for autism and early-life experience variables.

## Results

### Mental Health

Table 2 presents whether a PGS for autism relates to mental health outcomes. Without controlling for covariates, a one standard deviation higher autism PGS is associated with 0.11 units higher CES-D scores and increases in the odds ratio for reported depression by 1.46 and psychiatric problems by 1.54. After controlling for the covariates, these associations disappeared, though the coefficient of the autism PGS was still positive for self-reported depression. Non-significance of the autism PGS does not mean that it is unrelated to mental health outcomes given that covariates, such as education and marital status, might mask the effects of the autism PGS, and this might be influenced by behavioral signs of autism. The results also support that the autism PGS might be limited for predicting phenotypes with the average risk of autism considering a low prevalence rate in the population (15, 17). This suggests that correlations between the autism PGS and mental health outcomes might be more prominent among people with specific characteristics. Therefore, a subsequent analysis below focused on identifying who is more likely to have high- and low-risk mental health issues associated with the autism PGS.

**Table 2:** The correlation between autism polygenic score and mental health.

	**CES-D**	**Depression**	**Psychiatric** **problem**	**CES-D**	**Depression**	**Psychiatric** **problem**
	**Coef.** **(S.E.)** **[95% C.I.]**	**O.R.** **(S.E.)** **[95% C.I.]**	**O.R.** **(S.E.)** **[95% C.I.]**	**Coef.** **(S.E.)** **[95% C.I.]**	**O.R.** **(S.E.)** **[95% C.I.]**	**O.R.** **(S.E.)** **[95% C.I.]**
Autism PGS	0.1067*** (0.0163) [0.75, 0.14]	1.4598*** (0.0489) [1.37, 1.56]	1.5420*** (0.1313) [1.31, 1.82]	−0.0086 (0.0199) [−0.05, 0.03]	1.0281 (0.0458) [0.94, 1.12]	0.8338 (0.1232) [0.64, 1.11]
Controls	No	No	No	Yes	Yes	Yes
R-squared	0.0052	NA	NA	0.2154	NA	NA

*8,464 unique individuals, 34,536 observations*.

*** *p < 0.001*.

### Early Maternal Relational Quality

Table 3 shows the interaction of the early maternal relational quality and the autism PGS. Our results show that relational quality with mother before age 18 is correlated with better mental health. Specifically, a one standard deviation higher relational quality score related to 0.20 units lower CES-D scores and decreases in the odds ratio for depressions by 0.79 and psychiatric problem by 0.14. Further, this early life experience with the mother had a moderating effect on the relationship between the autism PGS and the odds of psychiatric problems. Although the coefficient of the autism PGS was not significant at *p* < 0.05 (but positive), the interaction between the two scores was still negative, suggesting that the early-life maternal relationship can be negatively associated with the increased risk of having psychiatric problems associated with the genetic risk for autism.

**Table 3:** Relational quality with mother as a moderator.

	**CES-D**	**Depression**	**Psychiatric problem**
	**Coef.** **(S.E.)** **[95% C.I.]**	**O.R.** **(S.E.)** **[95% C.I.]**	**O.R.** **(S.E.)** **[95% C.I.]**
Autism PGS	−0.0145 (0.0198) [−0.05, 0.02]	1.0199 (0.0453) [0.93, 1.11]	1.0019 (0.1586) [0.73, 1.37]
Relational quality with mother early in life	−0.1997*** (0.0146) [−0.23, −0.17]	0.7937*** (0.0251) [0.75, 0.84]	0.1445*** (0.0177) [0.11, 0.18]
PGS × Relational quality with mother	0.0041 (0.0148) [−0.02, 0.03]	0.9740 (0.0314) [0.91, 1.04]	0.6214*** (0.0752) [0.49, 0.79]
R-squared	0.2239	NA	NA

*Eight thousand four hundred and sixty four unique individuals, 34,536 observations*.

*** *p < 0.001*.

### Early Life Trauma and Discrimination

**Figure 1:**
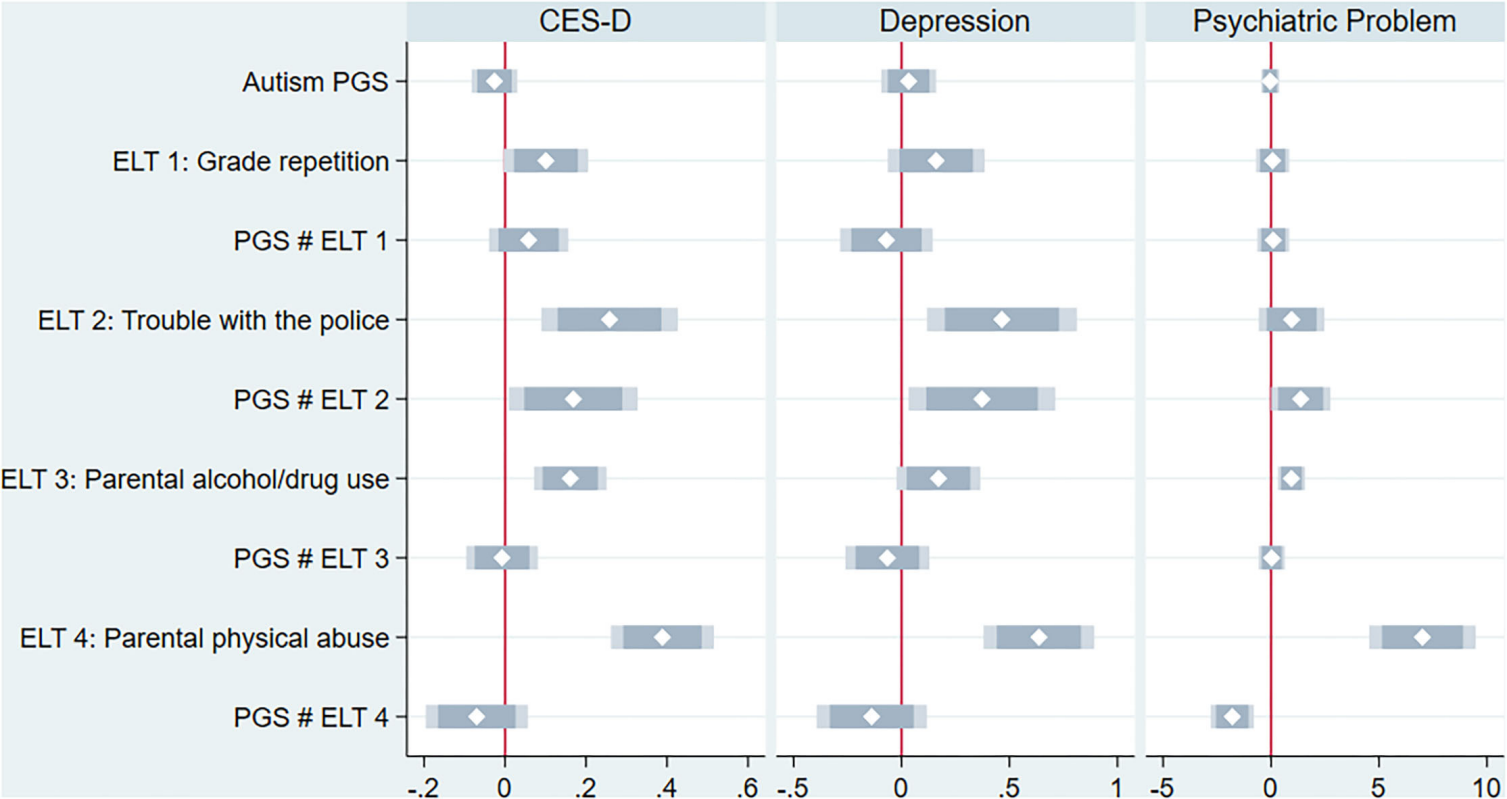
Early life trauma as a moderator between autism PGS and mental health.

Figure 1 shows the coefficients and their 95 and 99 percent confidence intervals of interaction terms between the autism PGS and four early life traumas (for the full results, see Supplementary Table 1). All four early life traumas were positively correlated with CES-D scores, indicating that those with early life traumas reported more negative feelings. Trouble with police, parental drinking/drug problems, and parental physical abuse were associated with an increased risk of depression and psychiatric problems. Of the four traumas, trouble with the police had an exacerbated effect on the correlation between the autism PGS and the three mental health outcomes. The effect of higher autism PGS and unpleasant experiences with police were related to increases in CES-D scores and the probability of depression and psychiatric problems.

**Figure 2:**
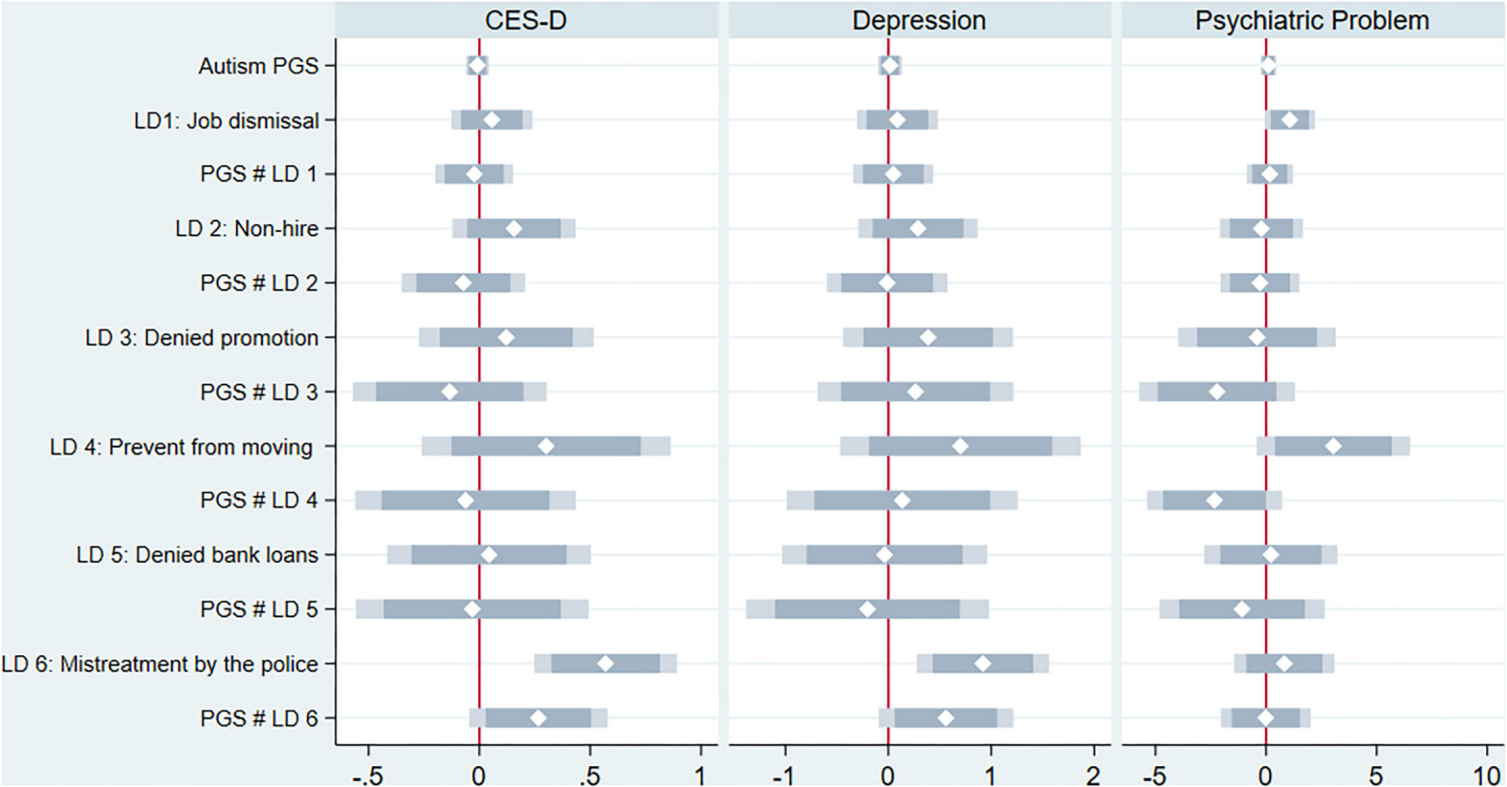
Early life discrimination as a moderator for the relation between autism PGS and mental health.

Figure 2 shows the estimates from the models testing of whether early life discrimination had a moderating effect on the correlation between autism PGS and mental health (for the full results, see Supplementary Table 2). Of the six discriminations, unfair treatment by the police was associated with increases in the CES-D score and the likelihood of depression—but not with psychiatric problems. More importantly, unfair treatment by the police had a moderating effect on the correlation between the autism PGS and the CES-D score and depression at *p* <0.05; that is, those with a higher genetic risk for autism and mistreatment by the police experienced poorer mental health later in life.

## Discussion

Using a nationally representative panel sample of the HRS, this study finds empirical evidence consistent with the diathesis-stress model. On average, an autism PGS does not have strong predictive power in explaining current and lifetime mental health after controlling for covariates. Our results, however, show differences in the likelihood of mental health issues among people with a higher autism PGS and particular early life experiences. Specifically, a stronger maternal relationship during childhood reduces the elevated likelihood of being diagnosed with psychological problems. Further, individuals with a high genetic risk of autism are more likely to suffer from current and lifetime mental issues if they had negative early life experiences, especially encounters with police. As shown in the diathesis-stress model, these findings underscore contextual factors such as early life experiences that are associated with the mental health of those with different levels of inherited susceptibility for psychiatric problems (12, 18).

It is important to note that the information obtained from the HRS participants is limited to data about the perceived quality of maternal relationships; i.e., participants are not asked about their relationships with other caregivers. However, quality relationships with caregivers in early life are associated with better mental health and fewer social communication difficulties of those with autism (1). Paired with the increased stress associated with being a caregiver of a child with autism (36), these issues may deter the development of high-quality child-caregiver relationships. To establish such relationships, interventions should support both children and their caregivers (37, 38). An activity-based approach to intervention (ABI) may be well-suited for providing simultaneous support to the child and caregiver. ABI focuses on embedding learning opportunities within daily routines and assists caregivers by identifying opportunities and learning strategies for interacting with the child within the context of a familiar environment (39, 40).

In addition to impacting the quality of relationships with caregivers, the social communication difficulties of children with autism may also affect encounters with other community members. Those with autism may be more likely to experience police encounters because of autistic characteristics that might be misinterpreted as odd or threatening (41, 42). These behaviors can also be mistaken by law enforcement officers as a lack of cooperation or strange behavior (42). There is a small but growing body of research that explores autism and police encounters. One study indicated that 20 percent of young people with autism have been stopped or questioned by police and 5 percent were arrested (43). Another study reported that 7.9 percent of those with autism were traumatized by police encounters (41). Since police encounters can be devastating for individuals with autism, further research is needed to identify the particular autistic characteristics that may increase the chance of police involvement (41, 42). Further, understanding the interplay between psychiatric problems, such as depression and police encounters, is critically important in the study of autism (41). Our results help to close this gap in the literature, by showing that individuals with a high genetic risk of autism are more likely to suffer from psychiatric problems when they encounter police in their youth.

To prevent such negative experiences, professionals have a role in interventions, facilitating communication for those with autism about law enforcement encounters. Helping them understand social conventions, emotional and verbal expressions, or the interpretation of the behavior of others are essential components of therapeutic goals (44). Therapists can further help autistic individuals avoid behaviors that might be considered inappropriate or suspicious, and lessen the stress and anxiety they might feel in unfamiliar encounters with law enforcement officers (44).

Despite the considerable emotional and financial costs for children with autism, their families, and society for police encounters (41), limited information is available on how police officers can respond to the needs of autistic individuals (42). The development of prevention programs and resources to create better awareness for law enforcement and communities is key to preventing distressing police encounters that might spark psychiatric problems, such as depression in later life (45).

The present study has several limitations. First, although a PGS for autism can serve as a relevant proxy for autism, the HRS does not ask whether respondents have been diagnosed with autism. For this reason, the study could not identify the prevalence of autism among its sample nor find any association between autism PGS and diagnosis. Although diagnoses with psychiatric problems (one of the outcome variables) can capture autism, the data prevent identifying those with autism at the time of the survey. Therefore, our results might be underpowered in quantifying autism risk in the general population that consist of people with the average level of risk (15, 17). In future research, the predictive power of the autism PGS could be improved if the HRS generates the score using convergent functional genomics that reduces statistical noise and prioritizes genes from SNPs after identifying them (46). Further, given that PGS is a measure of genetic risk and therefore does not provide information about diagnosis or symptom severity, the use of PGS as a proxy for autism may limit our ability to uncover an underlying mechanism for our findings. For example, although the findings indicate that early maternal relational quality is correlated with better mental health later in life, relying on genetic risk does not allow us to identify whether the moderating effect of early maternal relational quality systematically differs by autism symptom severity. Specifically, it is plausible that individuals with milder symptoms of autism and better early maternal relations show improved mental health later in life.

Despite these limitations, the present study contributes to the literature on autism in people by identifying *lifetime* effects of living with a genetic risk of autism and takes advantage of the HRS data, which enables the recovery of information about the respondents' early life events and history of psychiatric problems retrospectively. To our best knowledge, this is the first study that uses a *nationally representative longitudinal* (non-clinical) sample with *measured* genetic data on autism. Second, to mitigate biases that might occur by overlooking differences in genetic variances across ancestry groups, the study includes European descendants only. Although autism is more prevalent among non-Hispanic White groups than among Black and Hispanic groups (47), any future exploration of racial/ethnic differences might have significant implications for families of different cultural backgrounds.

To summarize, the present study contributes to the autism and mental health literature by identifying lifetime trajectories of mental health conditions with higher genetic risk for autism using a nationally representative panel sample based on the diathesis-stress model. It finds that early life experiences (i.e., relationship with mother, police encounters) are significant factors that mitigate or exacerbate the increased risk of suffering from current or lifetime mental issues for those who are genetically predisposed to autism. These findings provide important implications because participants in the study's sample (i.e., older adults) were unlikely to be diagnosed with autism but have a genetic propensity for autism. These findings could inform clinical and educational practices and provide implications for future research on the relationship between early life experiences and mental health for older adults with autism.

## Data Availability Statement

Publicly available datasets were analyzed in this study. This data can be found here: https://hrsonline.isr.umich.edu/.

## Ethics Statement

The University of Michigan IRB reviewed and approved the HRS involving human participants. The patients/participants provided their written informed consent to participate in this study.

## Author Contributions

SS cleaned the dataset and performed the statistical analysis. All authors contributed to conception of the study, wrote sections of the manuscript, and contributed to manuscript revision, read, and approved the submitted version.

## Conflict of Interest

The authors declare that the research was conducted in the absence of any commercial or financial relationships that could be construed as a potential conflict of interest.

## Publisher's Note

All claims expressed in this article are solely those of the authors and do not necessarily represent those of their affiliated organizations, or those of the publisher, the editors and the reviewers. Any product that may be evaluated in this article, or claim that may be made by its manufacturer, is not guaranteed or endorsed by the publisher.
