# Supplementary material for: Early Life Experiences Moderate the Relationship Between Genetic Risk of Autism and Current and Lifetime Mental Health
Source: Front Psychiatry. 2021 Nov 30;12:772841. doi: 10.3389/fpsyt.2021.772841 (PMC8669098; doi:10.3389/fpsyt.2021.772841)
Supplement: Supplementary file 1 [file Table_1.DOCX]

**Supporting Information**

S 1 Table. Early lifetime trauma as a moderator.

|  | CES-D | Depression | Psychiatric problem |
| --- | --- | --- | --- |
|  | Coef.  (S.E.)  [95% C.I.] | O.R.  (S.E.)  [95% C.I.] | O.R.  (S.E.)  [95% C.I.] |
| Autism PGS | -0.0261  (0.0218)  [-0.07, 0.02] | 1.0334  (0.0509)  [0.94, 1.14] | 0.9631  (0.1539)  [0.70, 1.32] |
| Early lifetime trauma 1: Did a year of school over again | 0.1010*  (0.0403)  [0.02, 0.18] | 1.1740  (0.1022)  [0.99, 1.39] | 1.0777  (0.3202)  [0.60, 1.93] |
| PGS × Did a year of school over again | 0.0581  (0.0380)  [-0.02, 0.13] | 0.9323  (0.0776)  [0.79, 1.10] | 1.1096  (0.3169)  [0.63, 1.94] |
| Early lifetime trauma 2: Was in trouble with the police | 0.2579***  (0.0654)  [0.13, 0.39] | 1.5915**  (0.2145)  [1.22, 2.07] | 2.5934  (1.5273)  [0.82, 8.23] |
| PGS × Was in trouble with the police | 0.1685**  (0.0617)  [0.05, 0.29] | 1.4510**  (0.1913)  [1.12, 1.88] | 3.9574*  (2.1124)  [1.39, 11.27] |
| Early lifetime trauma 3: Had parents drink or use drugs | 0.1610***  (0.0348)  [0.09, 0.23] | 1.1864*  (0.0893)  [1.02, 1.37] | 2.5671***  (0.6286)  [1.59, 4.15] |
| PGS × Had parents drink or use drugs | -0.0074  (0.0344)  [-0.07, 0.06] | 0.9364  (0.0706)  [0.81, 1.09] | 1.0326  (0.2419)  [0.65, 1.63] |
| Early lifetime trauma 4: Was physically abused by parents | 0.3887***  (0.0493)  [0.29, 0.49] | 1.8889***  (0.1879)  [1.55, 2.30] | 111.04***  (1062.12)  [170.17, 7240.86] |
| PGS × Was physically abused by parents | -0.0699  (0.0489)  [-0.17, 0.03] | 0.8711  (0.0864)  [0.72, 1.06] | 0.1662***  (0.0642)  [0.08, 0.35] |
| R-squared | 0.2215 | NA | NA |

*Note*. 8,464 unique individuals, 34,536 observations. * *p*<0.05, ** *p*< 0.01, *** *p*<0.001

S 2 Table. Early lifetime discrimination as a moderator.

|  | CES-D | Depression | Psychiatric problem |
| --- | --- | --- | --- |
|  | Coef.  (S.E.)  [95% C.I.] | O.R.  (S.E.)  [95% C.I.] | O.R.  (S.E.)  [95% C.I.] |
| Autism PGS | -0.0089  (0.0203)  [-0.05, 0.03] | 1.0168  (0.0460)  [0.93, 1.11] | 1.1106  (0.1516)  [0.85, 1.45] |
| Early lifetime discrimination 1: Unfairly dismissed from a job | 0.0559  (0.0710)  [-0.08, 0.19] | 1.0920  (0.1667)  [0.81, 1.47] | 2.9187*  (1.2874)  [1.23, 6.93] |
| PGS × Unfairly dismissed from a job | -0.0237  (0.0681)  [-0.16, 0.11] | 1.0485  (0.1582)  [0.7801, 1.41] | 1.1802  (0.4785)  [0.53, 2.61] |
| Early lifetime discrimination 2: Unfairly not hired for a job | 0.1559  (0.1079)  [-0.06, 0.37] | 1.3345  (0.2999)  [0.86, 2.07] | 0.8063  (0.5873)  [0.19, 3.36] |
| PGS × Unfairly not hired for a job | -0.0725  (0.1081)  [-0.28, 0.14] | 0.9888  (0.2248)  [0.63, 1.54] | 0.7554  (0.5229)  [0.19, 2.93] |
| Early lifetime discrimination 3: Unfairly denied a promotion | 0.1211  (0.1532)  [-0.18, 0.42] | 1.4713  (0.4718)  [0.78, 2.76] | 0.6628  (0.9160)  [0.04, 9.95] |
| PGS × Unfairly denied a promotion | -0.1341  (0.1697)  [-0.47, 0.20] | 1.3022  (0.4805)  [0.63, 2.68] | 0.1088  (0.1489)  [0.01, 1.59] |
| Early lifetime discrimination 4: Unfairly prevented from moving | 0.3011  (0.2181)  [-0.13, 0.73] | 2.0161  (0.9147)  [0.83, 4.91] | 20.8817*  (28.1208)  [1.49, 29.91] |
| PGS × Unfairly prevented from moving | -0.0627  (0.1932)  [-0.44, 0.32] | 1.1442  (0.4985)  [0.49, 2.69] | 0.0973*  (0.1152)  [0.01, 0.99] |
| Early lifetime discrimination 5: Unfairly denied a bank loan | 0.0435  (0.1784)  [-0.31, 0.39] | 0.9641  (0.3727)  [0.45, 2.06] | 1.2426  (1.4524)  [0.13, 12.28] |
| PGS × Unfairly denied a bank loan | -0.0324  (0.2039)  [-0.43, 0.37] | 0.8164  (0.3742)  [0.33, 2.00] | 0.3373  (0.4896)  [0.02, 5.80] |
| Early lifetime discrimination 6: Unfairly treated by the police | 0.5693***  (0.1249)  [0.32, 0.81] | 2.5059***  (0.6246)  [1.54, 4.08] | 2.2814  (2.0050)  [0.41, 12.77] |
| PGS × Unfairly treated by the police | 0.2660*  (0.1212)  [0.03, 0.50] | 1.7500*  (0.4444)  [1.06, 2.88] | 0.9856  (0.7758)  [0.21, 4.61] |
| R-squared | 0.2171 | NA | NA |

*Note*. 8,464 unique individuals, 34,536 observations. * *p*<0.05, ** *p*< 0.01, *** *p*<0.001
